# Gold Nanorod-Assisted Photothermal Therapy and Improvement Strategies

**DOI:** 10.3390/bioengineering9050200

**Published:** 2022-05-05

**Authors:** Mitchell Lee Taylor, Raymond Edward Wilson, Kristopher Daniel Amrhein, Xiaohua Huang

**Affiliations:** Department of Chemistry, The University of Memphis, Memphis, TN 38152, USA; mltylor3@memphis.edu (M.L.T.); rwlson14@memphis.edu (R.E.W.J.); kdmrhein@memphis.edu (K.D.A.)

**Keywords:** gold nanorod, photothermal therapy, cancer, surface plasmon resonance

## Abstract

Noble metal nanoparticles have been sought after in cancer nanomedicine during the past two decades, owing to the unique localized surface plasmon resonance that induces strong absorption and scattering properties of the nanoparticles. A popular application of noble metal nanoparticles is photothermal therapy, which destroys cancer cells by heat generated by laser irradiation of the nanoparticles. Gold nanorods have stood out as one of the major types of noble metal nanoparticles for photothermal therapy due to the facile tuning of their optical properties in the tissue penetrative near infrared region, strong photothermal conversion efficiency, and long blood circulation half-life after surface modification with stealthy polymers. In this review, we will summarize the optical properties of gold nanorods and their applications in photothermal therapy. We will also discuss the recent strategies to improve gold nanorod-assisted photothermal therapy through combination with chemotherapy and photodynamic therapy.

## 1. Introduction

In 2020, over 9 million people died of cancer and over 19 million new cancer cases were reported globally making cancer one of the deadliest diseases currently afflicting humankind [1]. Being a genetic disease characterized as an out-of- control proliferation of abnormal cells [2], cancer has been managed with novel treatments and more efficient early detection methods, but many of these treatments have serious side effects that require immediate mitigation [3].

Currently, mainstream treatment options are chemotherapy, radiotherapy, immunotherapy, and surgery. However, each of these treatments have significant shortcomings [4]. For example, due to the heterogeneity of cancer, chemotherapy, which is used to target quickly dividing cancerous cells, has the side effect of non-specific harm to normal cells [5]. Additionally, many chemotherapy drugs become ineffectual over time as cancerous cells quickly develop a resistance [6]. On the other hand, surgery, which is often used as a frontline defense for removing primary tumors, is invasive and can increase the risk of developing secondary cancers [7], while radiotherapy and immunotherapy can negatively impact the immune system [8,9].

To combat the complexity of cancer and the associated risks with current treatment options, attempts have been made to increase the efficacy and lower the safety risks of available therapeutic strategies. Among these attempts is photothermal therapy (PTT), a technique in which various sources of electromagnetic energy such as visible light, infrared, near infrared, radio waves, or microwaves, are used to heat localized areas of tissue in order to kill cancer cells via light-to-heat conversion [10]. However, due to the low absorption efficiency of tissue and the limited efficacy of many externally administered photothermal agents such as organic dyes, the adoption of PTT in clinical settings has been limited.

In the last two decades, the emergence of photothermally active nanoparticles have garnered new attention for PTT, reviving it as relevant strategy for non-invasive removal of cancerous tumors [4,11]. A number of nanoplatforms have been reported, including metal nanoparticles of various compositions, shapes and structures, carbon nanoparticles and nanotubes, graphene oxide, and some other inorganic nanoparticles such as copper chalcogenide [12,13,14,15,16,17,18,19,20]. The most popular type of NPs are near infrared (NIR)-absorbing AuNPs, including Au nanoshells, Au nanorods (AuNRs), Au nanocages, hollow Au nanospheres, and Au nanostars [21,22,23]. These nanoplatforms exhibit high photothermal properties, superior to previously used non- metal materials. The NIR light is beneficial for deep tissue penetration. Additionally, these particles have shown excellent biocompatibility and functionality, making them a viable alternative to other less effective externally administered agents. This gives rise to an enhanced form of PTT known as plasmonic photothermal therapy (PPTT) [24]. 

Of the existing NIR-absorbing Au nanoplatforms, AuNRs are especially intriguing for PTT due to their outstanding properties [25,26,27]. AuNRs are small, only about 40 nm in length and 10 nm in width [12]. However, the prototype Au nanoshells are more than 100 nm in diameter [28]. High quality AuNRs can be facilely prepared by the classic seed-mediated growth method [29,30], which is easier than the synthesis of Au nanoshells and nanocages. AuNRs exhibit excellent optical tunability by changing the aspect ratio (AR, length/width) of the particle [31]. Changing the AR can be facility achieved by changing the concentration of silver nitrate in the seed-mediated growth method [14]. It is also shown that AuNRs exhibit superior photothermal heat generation and blood circulation half-life (t_1/2_ = 17 h) [32] compared to Au nanoshells (t_1/2_ = 3–4 h) [33]. Furthermore, AuNRs can be easily conjugated with a plethora of bioactive and cancer killing species, opening the door for combination therapy such as PPTT + chemotherapy or PPTT + photodynamic therapy (PDT).

In this review, we start with a brief synopsis of the localized surface plasmon resonance (LSPR) property of AuNRs and their tuning by the dimension of the particles. This is followed by photothermal property that bases the PPTT. The PPTT is discussed by the tumor targeting mechanisms, passive and active tumor targeting. Combinational therapies with AuNRs are discussed based on the type of loaded drugs and are classified into PPTT + Chemotherapy and PPTT + PDT. The PPTT + chemotherapy is further discussed in terms of the mechanisms of drug releases that include mainly pH dependent, photo/thermo-responsive, and enzymatic drug release. At the end, we outline future direction of AuNR-assisted PPTT as well as barriers that need to be overcome for clinical applications. 

## 2. Optical Properties of Gold Nanorods (AuNRs)

### 2.1. Localized Surface Plasmon Resonance (LSPR)

As a metallic particle is reduced to a size comparable to or smaller than the electron mean free path (about 100 nm), the particle exhibits a unique photophysical phenomenon called localized surface plasmon resonance (LSPR) [25]. When electromagnetic radiation is shined onto such a plasmonic nanoscale metallic particle, the oscillating electromagnetic field interacts with the conduction band electrons of the particle, causing the electrons to oscillate coherently with the incident field and thereby inducing charge separation on the particle. At a specific frequency, the amplitude of the oscillation attains a maximum, thereby reaching plasmon resonance [34]. This resonance induces strong absorption and scattering (extinction) of the incident light, particularly when the NP consists of noble metals such as Au or silver (Ag). It is this resonance which confers the unique photothermal properties to AuNRs. 

The intensity of the LSPR band as well as the peak absorption wavelength depends on many physical properties of the NP, such as size, shape, charge density, and composition, as was first described by Gustav Mie in the early 1900s [35]. For example, Au colloid exhibits longer LSPR wavelength than silver colloid of the same size, whereas both metals demonstrate shorter wavelengths as the particle sizes are decreased [36]. Additionally, it is usually observed that Ag colloid yields higher scattering efficiencies than that of the same shape and size particles of Au colloid. Since physical characteristics play such important roles in LSPR, it is desirable and possible to design NPs of certain size, shape, and composition to have particular LSPR. For example, AuNRs are tunable in that their LSPR can be easily adjusted to desired wavelengths by altering certain synthesis parameters, such as the chemical concentrations of reagents [29,30,37]. This has important implications for PPTT as only certain wavelengths of light are effective for treatment, specifically in the NIR region. 

In traditional PTT, various wavelengths of light such has microwaves, visible, and UV are used since it is the tissue itself being targeted. However, in PPTT the incident light is directed onto the NPs themselves in order to heat the surrounding tissue [38]. Since these NPs are injected into the deep tissue, a wavelength of light must be chosen that will not be easily absorbed by the water and hemoglobin contained within the tissue, and must also possess enough energy to penetrate down to the NPs to irradiate them. The wavelengths of light fulfilling these requirements are in the NIR window ranging from approximately 700 nm to 2500 nm, where wavelengths ranging from about 700 nm to 1000 nm are typically used as tissue increasingly absorbs wavelengths starting at 950 nm [39,40,41]. By altering the physical dimensions of the AuNRs, UV radiation can more efficiently be absorbed or scattered by the particles.

**Figure 1:**
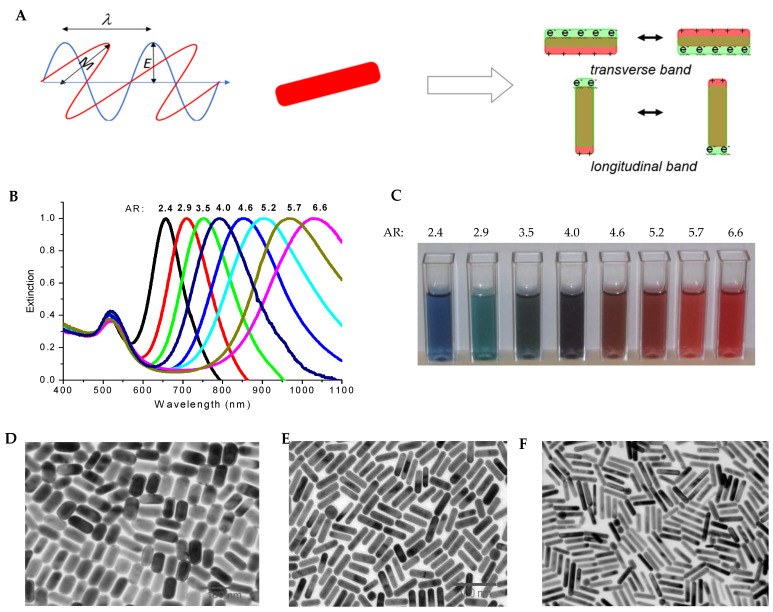
Optical Properties of AuNRs. (**A**) Schematic of the coherent and collective electron oscillations along the long and short axes of AuNRs resulting in longitudinal and transverse LSPR bands, respectively. Blue line is electric field and red line is magnetic field. (**B**) Dependence of LSPR of AuNRs on the aspect ratio (AR). Increasing AR leads to red shift of the longitudinal LSPR. (**C**) Photographic picture of the solution of AuNRs with different ARs. (**D**–**F**) TEM images of AuNRs with aspect ratio of 2.9 (**C**), 4.0 (**D**), and 4.6 (**E**).

AuNRs are inherently anisotropic, a characteristic which manifests as two distinct regions of extinction, known as the longitudinal and the transverse peaks that corresponds to the electron oscillations along the long axis and short axis, respectively (Figure 1). By varying the length of the AuNRs, different ARs can be generated which result in shifted UV–visible spectra [42]. Along the longitudinal axis, the surface plasmon oscillation results in an absorption band with a longer, redshifted wavelength as the AR is increased, while the transverse absorption band remains relatively unchanged. The optical behavior responsible for these effects has been well understood using Gans theory [43].

Adjusting the optical window for AuNRs can be accomplished using the seed-mediated synthesis approach [29,44]. In a typical procedure, 2–5 nm of AuNPs were synthesized by reduction of chloroauric acid (2 mL, 0.25 mM) with ice-cold sodium borohydride (120 μL, 10 mM) in the presence of 0.1 M cetrimonium bromide (CTAB). A 10 mL of growth solution containing 0.5 mM chloroauric acid, 0.1 M CTAB, and 80 μM silver nitrate was prepared, followed by the reduction of the chloroauric acid in the growth solution with ascorbic acid (0.14 mL, 80 mM). To the reduced growth solution, 14 μL of the seed solution is injected and AuNRs with AR of 3.5 will be formed within 2 h. By adjusting the concentration of silver nitrate in the growth solution, AuNRs with different ARs are formed, with higher concentration of silver nitrate leading to AuNRs of higher ARs. The mechanism of the formation of AuNRs has been demonstrated to be the symmetry breaking of small AuNPs by preferential deposition of Ag atoms or the binding of CTAB [29]. Symmetry breaking leads to the formation of stable Au {111} faces and less stable Au {100} faces. Thus, Au atoms are preferentially deposited onto Au {100} faces, leading to the formation of rod shape nanoparticles. 

Recently, Gonzalez-Rubio reported the novel synthesis of high quality AuNRs by disconnecting symmetry breaking from the seeded growth process [45]. The authors added n-decanol as the co-surfactant to prepare small AuNRs as the seeds. The use of n-decanol provides a more-rigid micellar system to make seeds in high yield. The growth of AuNRs from anisotropic small AuNR seeds greatly improves the reproducibility of AuNR synthesis by avoiding the irreproducibility during the symmetry breaking step using the 2–5 nm Au nanosphere as the seeds in the traditional synthesis. This new and robust method can make uniform AuNRs with LSPR from 600 nm to 1270 nm, allowing fine control of the dimension of AuNRs and the corresponding optical properties from NIR to far-red spectral window. 

### 2.2. Photothermal Property

Generating the heat necessary for the destruction of cancer cells via plasmonic NPs takes place through a series of photo-physical processes [46]. The formation of a hot metallic lattice by the absorbed light occurs via two distinct processes: electron–electron relaxation and electron–phonon relaxation. Extremely hot electron temperatures are achievable with these processes as temperatures can approach several thousands of degrees kelvin while lattice temperatures of a few tens of degrees can be attained. Heating of the local environment is then achieved through phonon–phonon relaxation of the lattice, which is the main route by which cancer cells can be destroyed or their functions dramatically altered. Tuning the AuNR LSPR bands allows for strong absorption of the incident light, resulting in more efficient heating of the local environment and therefore more efficient cancer cell destruction. 

The photothermal conversion efficiency (PCE), *η* is calculated using the following equation:

(1) $$\eta =\frac{hS\;\left(T_{max}-T_{amb}\right)-Q_{dis}}{I\left(1-10^{-A}\right)}\;\;\;\;\;\;\;\;\;\;\;$$

where *h* is the heat transfer coefficient, *S* is the surface area of the container, *T_max_* is the maximum equilibrium temperature, *T_amb_* is the ambient temperature of the surroundings, *Q_dis_* is a parameter expressing the laser-induced heat input by the container, *I* is the laser power, and *A* is the absorbance of the NPs at the laser wavelength [47]. *Q_dis_* (mW) is determined using water as the control by:

(2) $$Q_{dis}=103\;m\;C\;\Delta T/t$$

Kim et al. summarized the PCE of AuNPs of various shapes and structures including AuNRs, Au nanoshells, and Au nanocages [48]. AuNRs have a PCE of 50% or higher depending on the AR and the wavelength of the illumination laser [49,50]. The PCE of AuNRs is higher than Au nanoshells and comparable to Au nanocages [48].

## 3. Plasmonic Photothermal Therapy (PPTT)

One of the primary concerns of cancer is the spreading and invasion of cancer cells. These collections of abnormal cells essentially poison the surrounding healthy tissue by blocking nutrients and oxygen while allowing waste product concentrations to increase. As cancer progresses, cancerous cells from these tumors can become dislodged and migrate to other parts of the body in a process called metastasis. Oftentimes, these metastatic sites develop secondary tumors in which the cells resemble those of the primary tumor [51]. This process of metastasis and tumor formation is responsible for 90% of cancer related human deaths [52]. 

To target cancerous tumors effectively, therapeutics must have some specificity for tumor cells, otherwise the therapy will non-specifically attack surround healthy tissue resulting in no net benefit of the therapy. Traditional PTT uses light of various wavelengths to directly irradiate cancerous tissue. However, healthy tissue is damaged in the process as the therapy is inherently nonspecific [53,54]. This process allows the AuNRs to be targeted instead of direct irradiation of the tissue, resulting in more specific approach of PTT known as PPTT. 

### 3.1. PPTT with Passively Targeting AuNRs

In the late 1990s and early 2000s, several groups began demonstrating that various NPs could be taken up by cancerous cells and utilized for enhanced PTT [55]. However, it soon became clear that the circulation time and accumulation of NPs in cells needed to be improved. While AuNRs can be taken up by cells, this passive accumulation is limited due to the body’s natural defense mechanisms, specifically by phagocytic clearance through the reticuloendothelial system (RES) [56]. It is therefore necessary to develop methods that allow the AuNRs to evade the immune system.

**Figure 2:**
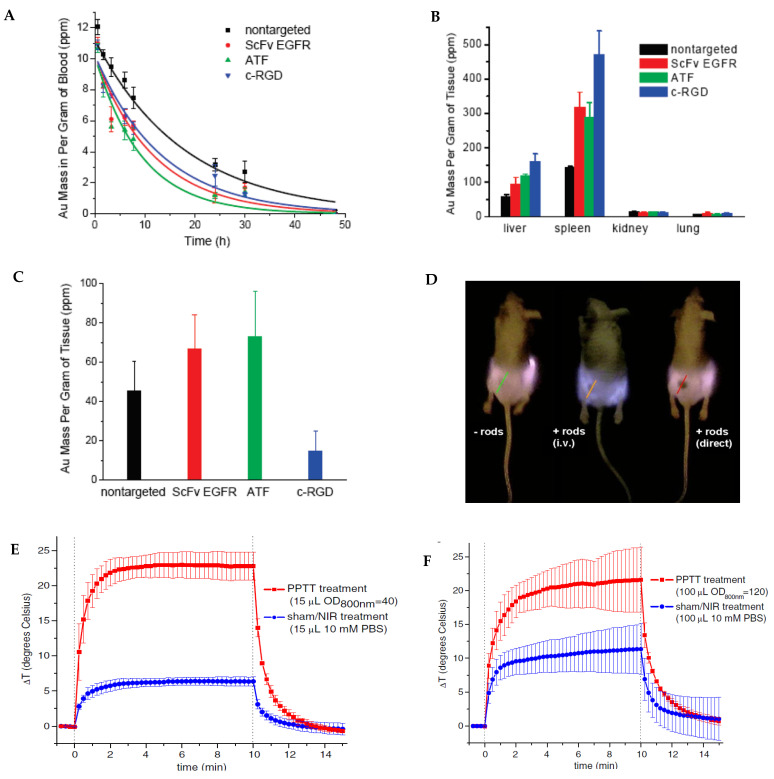

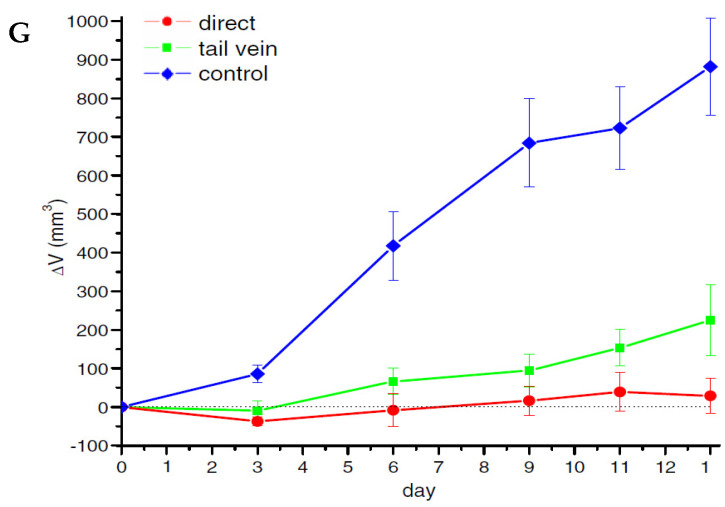
PPTT using passive targeting AuNRs. (**A**) Blood circulation for targeted and nontargeted AuNRs in healthy mice models. (**B**) Organ uptake of targeted and nontargeted AuNRs in healthy mice models at 24 h postinjection in A549 xenografted mice models. (**C**) Tumor uptake of targeted and nontargeted AuNRs at 24 h postinjection in A549 lung cancer xenografted mice models. AuNRs were quantified by ICP-MS in (**A**–**C**). (**D**) NIR transmission image of HSC-3 head and neck xenografted mice showing AuNRs in tumor for the intravenously and intratumoral administrated mice. (**E**,**F**) Thermal transient measurement of HSC-3 tumor interstitial during direct (**E**) and intravenous (**F**) NIR PPTT in comparison to sham/NIR treatment without PEGylated AuNRs. (**G**) Average change in tumor volume for HSC-3 xenografted mice following NIR PPTT by control (blue), intravenous (blue), and direct (red) injection of PEGylated AuNRs. (**A**–**C**) Reprinted/adapted with permission from Ref. [61]. Copyright © 2010, American Chemical Society. (**D**–**G**) Reprinted/adapted from Ref. [63]. Copyright © 2008, Elsevier.

Over the years, bioconjugation methods have been developed to ‘hide’ NPs to increase their retention time in the bloodstream as well as increase their concentration at tumor sites. One of the most successful methods of increasing the biocompatibility of AuNRs is PEGylation, which is to coat AuNPs with polyethylene glycol (PEG) [57]. In this method, the toxic stabilizing surfactant, CTAB that coats the AuNRs and helps maintain uniform dispersion of AuNRs, is replaced by a thiol–terminated PEG group. The retention time, cellular uptake, and overall biocompatibility of AuNRs are greatly enhanced due to the affinity of PEG for lipid membranes and their low immune response [58,59,60]. The studies by Huang et al. using inductively coupled plasma mass spectrometry (ICP-MS), showed that the blood half-life time for the PEGylatied AuNRs was 12.5 h, which is 3 to 6 h longer than AuNRs linked with active targeting peptides such as single-chain variable fragment of epidermal growth factor receptor (S_v_-EGFR), aminoterminalfragment (ATF), and cyclic arginine-glycine-aspartate (c-RGD) peptide [61] (Figure 2A). In contrast, the CTAB-capped AuNRs were cleared from the blood circulation within 15 min while the PEGylated AuNRs were mainly accumulated in spleen while the CTAB-coated AuNRs are accumulated in liver [62]. 

The research by Huang et al. also showed that the uptake of PEGylated AuNRs by RES organs such as liver and spleen was also significantly lower than AuNRs with targeting ligands S_v_-EGFR, ATF, or c-RGD peptide (Figure 2B) [61]. This is not surprising since the targeting ligands make the AuNRs less stealthy and more susceptible to clearance by the immune system. It is interesting to see that targeting ligands do not always have higher tumor uptake than PEGylated NPs. As a matter of fact, the AuNRs conjugated with c-RGD peptide had lower tumor uptake than the PEGylated AuNRs (Figure 2C). This is due to the much faster blood clearance and higher RES uptake of the c-RGD conjugated AuNRs than the PEGylated AuNRs. 

In 2008, Dickerson et al. demonstrated the feasibility of in vivo NIR PPTT with PEGylated AuNRs [63]. In this study, AuNRs with AR of 4.0 were modified with methoxy-PEG-thiol (mPEG-SH) with molecular weight of 5000. Mice bearing head and neck HSC-3 xenografts were administrated with PEGylated AuNRs either via tail vein or by direct injection into tumor. AuNRs inside tumors can be clearly visualized by NIR imaging due to the attenuation of light by the absorption of AuNRs (Figure 2D). For the treatment, tumors were exposed to NIR light (1.7–1.9 W/cm^2^, 6 mm in diameter) with a small and portable 808 nm diode laser for 10 min. Thermal measurements showed a temperature increase of tumor interstitial by over 20 °C for both direct and intravenous PPTT (Figure 2E,F). By monitoring tumor growth after treatment, the authors observed a >74% decrease in average tumor growth for intravenously-treated HSC-3 xenografts and a >96% decrease in average tumor growth for directly treated group at day 13 (relative to control tumors) (Figure 2G). Moreover, resorption of >57% of the directly treated tumors and 25% of the intravenously-treated tumors was observed over the monitoring period. The dramatic inhibition of tumor growth in the squamous cell carcinoma xenograft model suggests high clinical potential of PPTT with PEGylated AuNRs. 

Currently, PEGylation remain the most widely used surface functionalization for passive PPTT [64]. However, Chitosan, a natural polymer, is another popular biocompatible chemical with which to functionalize AuNRs, due to its ability to act as a backbone for thiomers excellent biocompatibility, biodegradable nature, cheap cost, and ease of modification using functional groups [65,66]. Additionally, chitosan can be used to increase specific tumor targeting as well as increase circulation time [39]. Choi et al. conjugated AuNRs with chitosan and found the particles displayed long circulation time and good tumor accumulation, as well as enhanced tumor targeting as opposed to AuNRs not conjugated with chitosan [67]. In their studies, the group incubated their chitosan coated AuNRs with tumor and fibroblast cells, which were then irradiated with 780 nm light of various power densities for 4 min. The cells were then stained with acridine orange and propidium iodide to characterize cell viability. The group found that the chitosan coated AuNRs accumulated mainly in the tumor SCC7 cells leading to enhanced cell death while the normal NIH/3T3 cells were not nearly as affected by the photothermolysis effect. To examine the in vivo effects of the chitosan coated AuNRs, the group injected the nanoparticles into the tail vein of athymic nude mice bearing bilateral SCC7 tumors. After 24 h, the tumors were excised and the distribution of AuNRs was analyzed using an inductively coupled plasma atomic emission spectrometer. The analysis revealed that the chitosan coated AuNRs had over 20% accumulation in the tumors whereas control PEG-modified AuNRs had only about 7% tumor accumulation. To analyze the therapeutic effect of the chitosan coated AuNRs, the researchers irradiated the mice with NIR laser radiation at 808 nm (4 W/cm^2^, 4 min) and found that no tumor growth was observed after 1 week of irradiation, thus demonstrating the effectiveness of chitosan coated AuNRs for photothermal therapy. 

Besides PEG and chitosan, other large or small molecules have been used in in the past decade, including mercaptopropionic acid, hydrogel, chondroitin sulfate A, and albumin [68,69,70,71]. In addition, research has been combing AuNRs with other materials such as fluorescent rare-earth nanoparticles and gold nanoclusters, graphene, mesoporous silica, and block copolymer to make nanocomposites to increase PPTT efficacy and or add diagnostic modalities [72,73,74,75,76,77]. 

Tissue penetration of the NIR light is critical for PPTT in vivo. A computational study by Cheong et al. using Monte Carlo simulations demonstrated the effectiveness of PPTT by tumor position and the type of tissue [78]. It was found that the type of tissue was more important than the distance of the tumor from the skin. Tissue with high scattering coefficient would reduce the photons to reach tumors. Tissue with high absorption coefficient would additionally cause heating of the tissue. Using bladder cancer as the disease model, the results show that PPTT was proven viable only when tumors was directly beneath the surface of the skin. When the tumors were positioned at the bottom half or at the side of the bladder, alternative treatments have been used to achieve effective treatment such as increasing laser power and cooling the skin to minimize heating of normal tissue. 

### 3.2. PPTT with Actively Targeting AuNRs

Specific cancer targeting is an inherently difficult task due to the heterogeneity of the disease. Thus, one of the primary goals of cancer therapeutics is the construction and effective implementation of target–specific therapies. Therefore, in addition to modify NPs to enhance their overall biocompatibility, intense research has been directed to develop methods to functionalize AuNRs with target–specific entities. Tumor–specific recognition molecules such as antibodies, folic acid, transferrin, and hyaluronic acid have been used with success by many groups [79,80,81].

**Figure 3:**
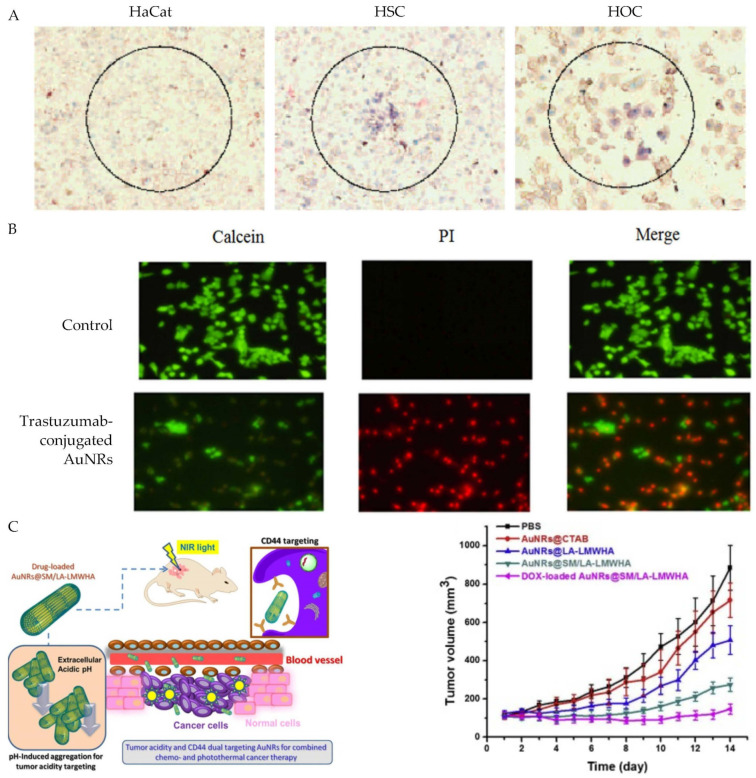
PPTT using ligand-conjugated AuNRs. (**A**) Selective PPTT of HSC (**middle**) and HOC (**right**) head and neck cancer cells over HaCat healthy cells (**left**) using anti-EGFR conjugated AuNRs. Reprinted/adapted with permission from Ref. [82]. Copyright © 2010, American Chemical Society. (**B**) PPTT of BT474 breast cancer cells with trastuzumab-conjugated AuNRs. Reprinted/adapted with permission from Ref. [85]. Copyright © 2017, Kang, X.; Guo, X.; An, W.; Niu, X.; Li, S.; Liu, Z.; Yang, Y.; Wang, N.; Jiang, Q.; Yan, C.; et al. (**C**) PPTT of MDA-MB-231 xenograft tumors with tumor acidity and CD44 dual targeting hyaluronic acid-coated AuNRs. (**Left**) Schematic of the targeting mechanism. (**Right**) Tumor volume at different days of post-treatment with 808 nm NIR laser and the hyaluronic acid-coated AuNRs. Reprinted/adapted with permission from Ref. [87]. Copyright © 2019, Elsevier.

In 2006, Huang et al. demonstrated for the first time the practicality of NIR PPTT, using AuNRs functionalized with anti-EGFR antibodies to specifically target EGFR-positive head and neck cancer cells in vitro [82] (Figure 3A). The AuNRs were synthesized to have a longitudinal extinction maximum wavelength at approximately 800 nm and then were incubated with anti-EGFR antibodies. Afterward, the anti-EGFR/AuNRs were incubated with head and neck cancer cells or nonmalignant epithelial cells for 30 min. Then, a continuous wave Ti:sapphire laser at 800 nm with a spot size of 1 mm was shined on the cancer cells for 4 min. Using this method, they found that the cancer cells were destroyed with half the laser energy (10 W/cm^2^) than the normal cells, demonstrating selective cancer cell therapy with PPTT and targeted AuNRs.

Since 2006, PPTT with active targeting AuNRs have been intensively investigated [83]. For examples, Turcheniuk et al. conjugated Tat protein to AuNR-reduced graphene oxide core-shell nanocomplex to target glioblastoma astrocytoma (U87MG) cells. Tumor suppression was observed upon low dose (0.7 W/cm^2^) of NIR light at 800 nm [84]. Kang et al. linked trastuzumab to AuNRs via porphyrin to target epidermal growth factor 2 receptor (Erb2 or HER2)-positive breast cancer. In addition to the targeted PPTT, the trastuzumab-AuNR complex also provide a targeted chemotherapy [85] (Figure 3B). Zhang et al. functionalized AuNRs with folic acid to target and treat melanoma cancer in a temperature-controlled manner [86]. Increasing temperature from 43 °C to 49 °C dramatically increased the fractions of dead cells. It also dramatically increased the proportion of cell death by necrosis from 10% to 50%.

To further increase the efficiency of cancer-specific targeting, Li et al. functionalized AuNRs with hyaluronic acid linked with pH-sensitive groups [87] [Figure 3C]. Hyaluronic acid has a preferential binding for the CD44 glycoprotein receptors that are overexpressed on many types of cancer cells [88]. Thus, the AuNRs preferentially accumulated into the acidic tumor sites and then were selectively uptaken by CD44-positive cancer cells. This dual tumor acidity and CD44 targeting not only benefits PPTT, but also chemotherapy that requires intracellular uptake of chemotherapeutic agents. DNA has also been successfully functionalized onto AuNRs via ligand exchange mechanisms which greatly improves the biocompatibility and functionality of the NPs [89]. Other groups have successfully immobilized DNA onto AuNRs along with various cancer drugs due to the ability of many drugs to bind DNA by intercalation [79]. For example, Wang et al. designed doxorubicin (DOX) loaded AuNRs by first coating the nanorods with calf thymus deoxyribonucleic acid via electrostatic interaction, then mixing the rods with DOX. The group found that the DNA/DOX AuNRs displayed good biocompatibility as well as enhanced toxicity vs. free DOX, due to the preferential uptake of the AuNRs by treated 4T1 mammary cancer cells. Up to date, many other ligands have been used for active PPTT with AuNRs, including RGD, folate, chondroitin sulfate, zwitterionic stealth peptide, and most of them were used to target surface protein markers on tumor cells [90,91,92,93,94].

### 3.3. Temperature Distribution of PPTT In Vitro and In Vivo

To effectively treat cancer, the temperature control and distribution in cancerous tissues are critically important. Heat transport in tissues have been studied for decades and it is now mainly based on the application of Penne’s equation [95]. A number of theoretical and simulation studies have been performed to investigate the temperature distribution of photothermal nanoparticles under laser irradiation in vitro and in vivo. For examples, Huang et al. theoretically studied the spatiotemporal temperature distribution of AuNR solution in a well that was irradiated with a laser using the Penne’s bioheat equation with an additional term counting energy release by nanoparticles under laser irradiation [96]. The model predicted that the highest temperature was located along the well axis where the laser is focused while the coolest temperature was near the wall at the surface of the fluid, in agreement with experimental results. The authors further studied both experimentally and theoretically the cell death by irradiating AuNR solution on top of cell monolayer. They found that the extracellular hyperthermia is effective in killing cancer cells, rationalizing the effectiveness of passive PPTT when AuNRs are located out of cancer cells in tumors. 

To understand the minimum temperature required for cell destruction, Huang et al. predicted the temperature rise on the basis of a numerical model [97]. The model considered both heat generation by the laser irradiation of the nanoparticles and heat depletion into the surroundings by conduction. These two processes were modeled into a temperature calculation algorithm using Fourier heat equation. The studies predicted the nanoparticle concentration and laser power in order to induce a defined temperature to kill cancer cells. It was estimated that the temperature was raised to 73–77 °C for gold nanoparticles with optical density (OD) of 0.159 with laser power of 150 mW. To reach the same temperature for the gold nanoparticles with OD of 0.03, the laser power needs to be increased to 450 mW. 

The studies by von Maltzahn et al. have helped us to understand the temperature distribution of AuNR-assisted PPTT in the tumor model [30]. They simulated temperature distribution of injected PEGylated AuNRs in tumor by finite element modeling as well as temperature on tumor surface and various depths in tumor for intravenously injected PEGylated AuNRs and saline mice. The studies showed that tumor surface temperature increased to 70 °C, matching experimental data. They also predicted that the entire tumor volume would be heated to over 60 °C at 5 min after laser irradiation (2 W/cm^2^). These computational studies well explained the effectiveness of tumor ablation using PPTT and AuNRs. A more thorough computational study was performed later by Kannadorai and Liu using the Pennes equation and first-order thermal-chemical rate equation to model the temperature and thermal damage distributions in spherical tumors with AuNRs [98]. This method helps optimize nanoparticle concentration, laser power density, and exposure time for PPTT of deep-seated tumors. Differently, Manuchehrabadi et al. used Monte Carlo method to simulate the temperature rise of PPTT in tumors. Their results showed that permanent thermal damage occurs in the tumor injected with the 250 OD AuNR solution after heating for 15 min while 50 OD AuNR would not completely eradiate tumor. 

### 3.4. Mechanisms of PPTT

PTT causes cell damage by hyperthermia. However, the photothermal activities of AuNPs in PPTT extend beyond simple hyperthermia. The studies by Tong et al. in 2007 laid a foundation for understanding cell death for PPTT. They incubated KB cells with folate-conjugated AuNRs, treated the cells with NIR irradiation, and monitored cell death in real-time with two-photon luminescence (TPL) and bright field imaging [99]. They found that AuNRs mediated cancer cell death by compromising membrane integrity and cavitation. The membrane perforation led to influx of Ca^2+^, followed by degradation of actin network. This induced dramatic blebbing of plasma membrane, which was observed within seconds after laser irradiation. The Ca^2+^ influx also induces injury of mitochondria, as evidenced on macrophage cell damage after PPTT [100]. Huang et al. also found that the efficacy of photothermolysis of cancer cells depends on the location of the AuNRs. The AuNRs on cell membrane are 10 times more effective than AuNRs inside cells in cell destruction. Depending on the type of laser, PPTT caused cell death either by apoptosis or necrosis. The continuous wave laser has been found to cause cell death by apoptosis while nanosecond pulsed laser led to cell necrosis [101].

Mechanistic studies of AuNRs in vivo were conducted in 2017 by Ali et al. using proteomic analysis in mouse tumor tissues [102]. The authors firstly optimized the concentration (2.5 nM), laser power (2 W/cm^2^), and surface ligands (rifampicin) to achieve maximal induction of apoptosis of tumor cells. The proteomic analysis revealed several death pathways mainly the apoptosis and cell death by releasing neutrophil extracellular traps (NETs) that were contributed by Pin1 and IL18-related signaling. Cytochrome c and p53 related apoptosis were observed to contribute the enhanced PPTT effect by the ligand conjugated AuNRs. Furthermore, they found that AuNRs aggerated after PPTT by differential interference contrast microscopy, due to the release of surface ligands after laser irradiation. Certainly, the aggregation of AuNRs alters the optical property and thus the phothermal efficacy by the particles. The combined effects of active tumor cell targeting and particle aggregation contributed the more effective treatment with ligand-conjugated AuNRs than passive PEGylated AuNRs. Their studies further showed that the AuNRs did not induce toxicity in vivo during the 15-month of study time, indicating long-term safety of AuNRs for PPTT. 

## 4. PPTT + Chemotherapy

The ability to make biocompatible AuNRs and functionalize them with a plethora of bioactive, specific tumor targeting agents has spurred researchers to further improve PPTT via combination with chemotherapy. Many groups have demonstrated dramatic improvements in the efficiency of chemotherapy by combining the temperature effects of PPTT with the ability of AuNRs to carry many drugs and specifically transport them to tumor sites. These studies have shown that combinatorial PPTT + chemotherapy greatly improves chemotherapy efficacy. Additionally, specific drug–release mechanisms have also been engineered for many different types of nanoparticles including AuNRs, making them not only being able to transport chemotherapeutic drugs to specific locations, but also function as controlled drug–release devices. To achieve effective chemotherapy, drug molecules need to be released from AuNRs in order to enter cells and nucleus. Three major drug release mechanisms have been used in PPTT + chemotherapy and they are pH, photo/thermo, and enzyme responsive mechanisms.

### 4.1. pH Dependent Drug Release

It is typically found that within the environment of cancer cells, the pH ranges between 6.4 and 7.0 while the environment of normal cells ranges from around 7.2 to 7.4 [103]. The difference in the two distinct cellular environments makes it possible to design chemical drug–release mechanisms for these specific cellular environments, typically by either chemical cleavage or a change in charge between chemical species.

**Figure 4:**
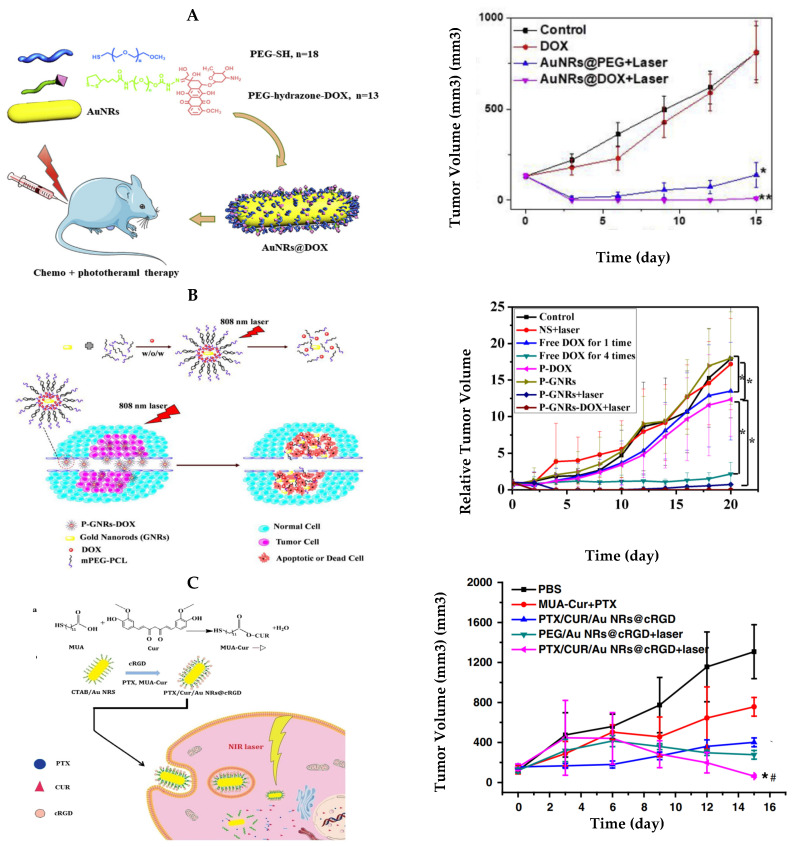
PPTT + chemotherapy using AuNRs. (**A**) Dox-conjugated pH-responsive AuNRs for PPTT + chemotherapy. **Left**: Schematic of the synthesis of the AuNR conjugates and its application for combined PPTT and chemotherapy. **Right**: Average HepG2 tumor volume of the mice at different days after different treatments. Cited from Ref. [104]. * indicates significant difference at *p* < 0.05 and ** indicates significant difference at *p* < 0.01. Copyright@2018, Chen, J.; Li, X.; Zhao, X.; Wu, Q.; Zhu, H.; Mao, Z.; Gao, C. (**B**) Dox-conjugated light-sensitive AuNRs for PPTT + chemotherapy. **Left**: Schematic of the preparation of P-GNRs-Dox and light sensitive drug release mechanism. **Right**: Relative C26 tumor volume of the mice at different days after different treatments. * indicates significant difference at *p* < 0.05. Cited from Ref. [108]. Copyright @2015, Liao, J.; Li, W.; Peng, J.; Enzu, Q.; Li, H.; Wei, Y.; Zhang, X.; Qian, Z. (**C**) Paclitaxel (PTX)/CUR/AuNRs@c-RGD complex for PPTT + chemotherapy. **Left**: Schematic of the preparation and application of the complex for PPTT + chemotherapy. PTX is released by hydrophobic interaction and CUR is released by enzymatic hydrolysis. **Right**: Average A549 tumor volume of the mice at different days after different treatments. Cited from Ref. [113]. * indicates significant difference at *p* < 0.05. * *p* < 0.05 vs. PTX/CUR/AuNRs@CRGD and # *p* < 0.05 vs. PEG/AuNRs@cRGD+laser. Copyright@2019, Zhu, F.; Tan, G.; Zhong, Y.; Jiang, Y.; Cai, L.; Yu, Z.; Liu, S.; Ren, F.

A common pH responsive method for the delivery of DOX into cancer cells is through a cleavable hydrazone linkage. In this method, hydrazine is coated onto AuNRs and is able to react with DOX via the ketone groups of the drug, thus creating a cleavable AuNR–hydrazone–DOX linkage. The linkage is then cleaved once it enters the lower pH cancerous environment due to the pH sensitive bond. Chen et al. constructed DOX carrying AuNRs via a-lipoyl-u-doxorubicinyl PEG with a hydrazone linker (LA-PEGHyd- DOX) [104] Figure 4A). They found that in a cellular pH environment of 7.4, only 17% of DOX was released after a 24-h incubation time. However, 90% of DOX was released in the lower pH cancerous environment (pH = 4.5) during the same time interval. During their in vitro experiments, they demonstrated that human liver carcinoma cells (HepG2) treated with the AuNRs@DOX displayed a dead cell population of >60% upon laser irradiation while their control AuNRs@PEG treatment displayed only 30%, confirming the benefit of combinatorial therapy.

In another study, Abbasian et al. constructed pH sensitive AuNR–cored biodegradable micelles by first coating AuNRs with a triblock copolymer containing a thiol end group [105]. A solution containing the conjugated AuNRs was incubated with DOX for 48 h at room temperature where the DOX was integrated into the polymer via the electrostatic force. Then, they conducted a series of in vitro pH responsiveness tests of the DOX functionalized AuNRs in pH solutions of 4, 5.4, and 7.4 where they found the percentage of drug release was 70, 50.8, and 22%, respectively, demonstrating the pH dependent release of DOX. In their cytotoxicity tests, the group found that their AuNRs@polymer decreased the viability of MCF7 breast cancer cells to about 65% when laser irradiated, while their AuNRs@polymer/DOX reduced the cell viability to about 21% upon laser irradiation. Furthermore, when AuNRs@polymer/DOX were not irradiated, the cell viability was about 55%, demonstrating the improved effects of the combinatorial PPTT and DOX treatment.

### 4.2. Photo/Thermo-Responsive Drug Release

Many polymers are thermoresponsive in that they can either be shrunk when heat is applied [106,107], or they can become degraded if their melting point is lower than the local temperatures produced upon irradiation. Drugs can be encapsulated within polymers conjugated to AuNRs and then released once the AuNRs are irradiated after reaching a cancerous target. For example, Liao et al. designed AuNRs encapsulated by an amphiphilic block polymer which contained an appropriate hydrophilic/hydrophobic ratio to self–assemble into polymersomes [108] (Figure 4B). DOX was inserted into the polymersomes due to the amphiphilic nature of the polymer, then the AuNRs and Dox co-loaded polymersomes (P-GNRs-DOX) were incubated with C26 tumor cells. Upon irradiation by an NIR laser, the group observed a 73.22% reduction in live cells, while only a 57.48% reduction in live cells was observed when the P-GNRs-DOX were not irradiated by the laser. One time treatment with P-GNRs-DOX and laser irradiation completely eradicated tumor in the C26 mouse model. In contrast, chemotherapy with free DOX after four dosages still did not completely inhibit tumor growth. PPTT alone did not completely destroy tumors, either, since 30% of the mice grew new tumors after 14 days. Thus, the P-GNRs-DOX, which only requires a single light irradiation, is very promising for cancer therapy by combined PPTT and chemotherapy with a light-activated drug release mechanism.

DNA based self-assembled targeted NIR responsive AuNRs were developed by Xiao et al. [109]. The platform consisted of 3 distinct functional components: complementary DNA strands for drug transport, photothermally active AuNRs, and PEG layer to evade the immune system. DOX was used for the chemotherapy agent, which was able to bind to the DNA by intercalation, and the amount of drug loading was controllable by adjusting the number of complementary DNA strands. Additionally, one strand of the DNA (termed the capture strand) was thiolated which allowed it to be anchored to the surface of the AuNRs. The other complementary strand (termed the targeting strand) was constructed by N-hydroxysuccinimide (NHS) terminated (TCG) oligonucleotide (ONT) with NH_2_-termniated PEG-folic acid for cancer cell targeting. Upon NIR irradiation, DOX was able to be release from the AuNRs as light causes complimentary DNA strands to unwind.

In their in vivo studies, the group demonstrated significantly higher tumor reduction using the functionalized AuNRs containing DOX vs. the control group contain no DOX.

Tang et al. developed “smart” polymer shell functionalized AuNRs responsive to heat/NIR by using poly(N-iso-propylacrylamide) (PNIPAM) which, through a phase transition, was able to change from a hydrophilic, water swollen state to a hydrophobic state [110]. First, AuNRs were modified by a mesoporous silica coating, yielding a “shelled” AuNR, then PNIPAM was added to the AuNRs@SiO_2_ which allowed DOX to be absorbed into the complex. The group found that at 37 °C the release percentage of DOX was only 10% after 24 h, however, increasing temperature yielded higher DOX release percentages. At 43 °C for example, it was found that 70% of the DOX has been release from the nanoparticles, demonstrating the temperature–dependent release of DOX.

### 4.3. Enzymatic Drug Release

It has been shown some enzymes are over expressed in cancerous environments [111], and since enzymes are able to catalyze the breaking of specific cleavable chemical bonds, another opportunity for specific cancer targeting and drug release is possible through the usage of enzymes [112]. Zhu et al. loaded PTX and chemically conjugated curcumin (CUR) onto AuNRs with an 11-mercaptoundecanoic acid (MUA) linker. Then, they conjugated with a c-RGD peptide specific to target the α_v_β_3_ integrin receptor overexpressed on the surfaces of some tumor cells [113] (Figure 4C). Based on their design, the release behavior of PTX and CUR should have no influence on each other since CUR is released by enzymatic hydrolysis through the engineered CUR chemical bonds and PTX is released by hydrophobic interaction with cell membrane. To test this hypothesis, the group performed a series of in vitro experiments where they found in the presence of esterase, an enzyme known to be over expressed in some cancer cells, CUR was released in increasing amounts as the esterase concentration was increased. On the other hand, PTX demonstrated no obvious release under the same conditions. However, under the environment of an imitated lipophilic plasma membrane, they found that more than 70% of the loaded PTX was released while almost none (<5%) of the CUR molecules was released, demonstrating independent dual release mechanism for the drug-loaded AuNR complex.

Another example is the studies by Liu et al. who created functionalized AuNRs with a two-stage sequence disassembly property, allowing for greater tumor accumulation and penetration [114]. The platform was constructed with amphiphilic block copolymers and the reduction-responsive prodrug DOX which was conjugated to the AuNRs by disulfide -linked poly(acrylic acid). Essentially, a PEG shell surrounded the DOX–coated AuNRs, thus during the first stage of release the AuNRs were able to avoid the immune system and retain a high circulation time resulting in greater tumor accumulation efficiency. In the next stage, the PEG shell was removed and the DOX–coated AuNR clusters were able to assemble into single entities, thereby more able to penetrate to the solid tumor. The group found that after the AuNRs entered the more acidic environment of a tumor, high concentrations of naturally glutathione present were reduced, and thus DOX was released into the tumor environment in significant amounts.

### 4.4. Other Release Mechanisms

In 2014, Huang’s group developed a PPTT + chemotherapy method with drug release through partitioning of hydrophobic PTX on AuNR within the lipophilic plasma membrane [115]. In this work, PTX was loaded to AuNRs with high density (2.0 × 10^4^ PTX per AuNR) via nonspecific adsorption, followed by stabilization with PEG linked with MUA. PTX was entrapped in the hydrophobic pocket formed by PEGy-MUA on the surface of AuNRs, which allows direct cellular delivery of the hydrophobic drugs via the lipophilic plasma membrane. Combined PPTT and chemotherapy with the PTX-loaded AuNRs was shown to be highly effective in killing head and neck cancer cells and lung cancer cells, superior to photothermal therapy or chemotherapy alone due to a synergistic effect.

Currently, PPTT + chemotherapy remains popular due to the combined advantages of the two treatments [116,117,118,119,120,121,122,123,124,125,126,127,128,129,130,131,132,133,134,135,136,137,138]. While PPTT can quickly eradicate tumor without side effect, the chemotherapy can eradicate tumor residue or metastasized cancer cells that are inaccessible by laser irradiation. The two together generally have synergistic effect to augment therapeutic efficacy. This is due to the fact that the AuNR-enabled laser heating can generally increase cellular uptake of chemotherapeutic drugs. 

## 5. PPTT + Photodynamic Therapy (PDT)

Another combinatorial therapy that has gained interest over the last few years is PPTT with PDT. PDT relies on a unique species called photosensitizer (PS) that is excited after absorbing incident photon energy. The energy from this excited state (electrons) is then transferred to oxygen molecules to produce high energy reactive oxygen species (ROS) to kill cancer cells.

**Figure 5:**
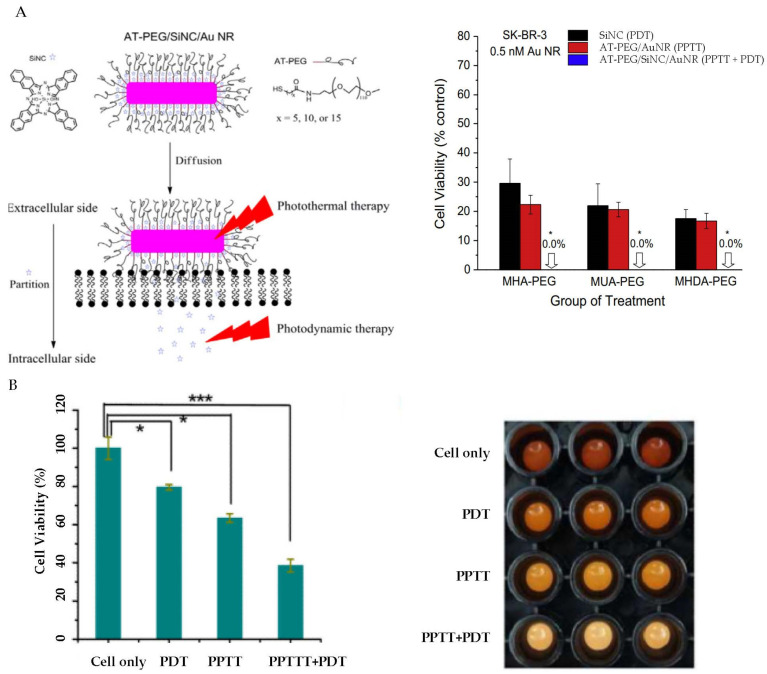
PPTT + PDT using AuNRs. (**A**) AT-PEG/SiNC/AuNRs for PPTT + PDT. **Left**: Schematic of the structure of AT-PEG/SiNC/AuNRs and mechanism of PPTT + PDT. **Right**: Comparison of SK-BR-3 cell viability data under different treatments. Reprinted/adapted from Ref. [139]. * indicates significant difference at *p* < 0.05. Copyright@2016, Elsevier. (**B**) Ce_6_-ASP-T_32_- NRs for PPTT + PDT. Comparison of CCRF-CEM cell viability data (**left**) and imaging under different treatments. * and *** indicates significant difference at *p* < 0.05 and *p* < 0.0001, respectively. Reprinted with permission from Ref. [142], Copyright © 2012, American Chemical Society.

In 2016, Bhana et al. reported a method to achieve combined PPTT and PDT by making a silicon 2,3-napthalocyanine dihydroxide (SiNC)/AuNR complex [139] (Figure 5A). In this study, SiNC was adsorbed onto AuNRs, followed by a stabilization via covalent binding of alkylthiol linked PEG (AT-PEG). Their studies showed that the SiNC loading efficiency depended on the AT chain length. Increasing the chain length of AT resulted in an increase in loading efficiency, but a decrease in release rate. The combined PPTT and PDT resulted in 17.3% cell viability for KB-3-1 cells and 18.3% for SK-BR-3 cells. However, the cell viability was much higher under PPTT alone, with 40.7% for KB-3-1 cells and 33.9% for SK-BR-3 cells. The cell viability for PDT alone was also much higher, with 58.4% for KB-3-1 cells and 59.9% for SK-BR-3 cells. This study clearly showed that the PPTT and PDT combination treatment was much more effective in killing cancer cells than PPTT or PDT alone.

Aptamers have been used in many biotechnology applications, especially as molecular switches [140,141]. Aptamers can also be used in PPTT and PDT. Aptamer switch probe (ASP)–modified AuNRs were designed by Wang et al. in which AuNRs were constructed to transport chlorin e6-polyvinylpyrrolidone (Ce_6_-PVP) to target leukemia cells [142] (Figure 5B). When the nanosystems came into contact with cancer cells, the ASP changed confirmation, thereby releasing Ce6 from the surface and thus creating ROS and destroying cancer cells. The ROS generation is controlled by varying the distance between a quencher and the photosensitizer via aptamer switches, effecting the quenching and recovery of photosensitizer fluorescence. Upon white light irradiation, ROS are generated leading to cancer cell death. The group found that treatment of cells by PDT alone resulted in 80% cell viability, whereas under PPTT alone resulted in a cell viability of 63%. However, PPTT + PDT therapy resulted in a cell viability of 40%, demonstrating the effectiveness of their combinatorial nanosystem.

Other PS agents have also been used for PPTT + PDT with AuNRs [143,144,145,146,147]. For examples, Jin et al. combined methylene blue with AuNRs in a hydrogel to form a PPTT + PDT complex with multiple advantages including excellent cancer cell ablation efficiency, controllable mechanical properties, excellent stability, and good cytocompatibility [146]. In the studies by Tian et al., platinum-based metallacycle-core star polymers were combined with AuNRs to achieve triple therapy of PPTT, PDT, and chemotherapy [147]. 

## 6. Conclusions and Outlook

AuNRs have thus shown outstanding optical properties suitable for NIR PPTT. They have tunable LSPR in the NIR window by controlling the AR of the particles, with photothermal conversion efficiency 50% and above. Intensive in vitro and in vivo studies have demonstrated their potential for future clinical use. By manipulating surface chemistry and drug release mechanism, AuNRs can carry a variety of chemotherapeutic drugs for combinational PPTT and chemotherapy. A number of drug release mechanisms have been utilized, including pH, photo/thermal, enzyme, and partition-based drug releases. This combined PPTT and chemotherapy has dominated current research interests in the field of nanotherapeutics using AuNRs. AuNRs can also carry photosensitizers for simultaneous PPTT and PDT under a single NIR irradiation. It is now clear that these combinational treatments are more effective than individual treatments alone, with or without synergistic effects. 

An emerging new direction for PPTT is to combine PPTT with immunotherapy to eradicate primary tumors while treating and inhibiting metastasis [148,149,150,151,152,153,154,155,156,157]. Cancer metastasis is the major cause of cancer death. Preventing or treating cancer metastasis would largely increase patient survival. The work by Chen et al. presented an early attempt to treat metastasized cancer by combining PPTT with AuNRs and checkpoint-blockade immunotherapy [148]. This was carried out by co-encapsulating imiquimod (R837), a Toll-like-receptor-7 agonist with AuNRs using poly(lactic-co-glycolic) (PLGA) acid. The nanoparticles not only ablated primary tumor, but also generated tumor-associated antigens to give vaccine-like functions. The generated immunological responses attacked remaining tumor cells in mice for metastasis inhibition. Using the combination of PPTT with immune-checkpoint inhibition, Liu et al. achieved synergistic treatment and completely eradicated primary tumor cells and distance untreated tumors in some mice with bladder cancer [150]. In the studies by Nam et al., the immunotherapy for disseminated tumors was triggered by combined PPTT and chemotherapy [152]. The PPTT alone can also trigger immune response to prevent metastasis by perturbating metastasis-related pathways [156]. This PPTT-induced vaccination has high promises to address the challenges in treatment of late stages of cancer whereas PPTT is inaccessible and chemotherapy has limitations due to drug resistance. It is expected that this PPTT-immunotherapy will be the next generation of cancer therapy using photothermal nanoparticles [157]. 

Nonetheless, several parameters remain to be addressed before approval by FDA for clinical use. As with other hard inorganic nanomaterials, AuNRs are not biodegradable. Although AuNRs are biocompatible, the long-term toxicity is not known. As the biodistribution studies have shown that majority of AuNRs are uptaken to liver and spleen. New strategies are needed to enhance the efficiency of tumor accumulation in order to further improve the therapeutic efficacies. Further, mechanisms for tumor extravasation, fate of AuNRs after treatment, and interactions with blood components in circulation remain to be investigated and understood well before use on patients.

## Data Availability

Not applicable.
